# Does C-Reactive Protein Contribute to Atherothrombosis Via Oxidant-Mediated Release of Pro-Thrombotic Factors and Activation of Platelets?

**DOI:** 10.3389/fphys.2012.00433

**Published:** 2012-11-16

**Authors:** Zhuo Zhang, Yan Yang, Michael A. Hill, Jianbo Wu

**Affiliations:** ^1^Drug Discovery Research Center, Luzhou Medical CollegeLuzhou, China; ^2^Dalton Cardiovascular Research Center, University of MissouriColumbia, MO, USA; ^3^Department of Medical Pharmacology and Physiology, University of MissouriColumbia, MO, USA

**Keywords:** atherothrombosis, C-reactive protein, platelet, reactive oxygen species, vascular cells

## Abstract

Inflammation and the generation of reactive oxygen species (ROS) have been implicated in the initiation and progression of atherosclerosis. Although C-reactive protein (CRP) has traditionally been considered to be a biomarker of inflammation, recent *in vitro* and *in vivo* studies have provided evidence that CRP, itself, exerts pro-thrombotic effects on vascular cells and may thus play a critical role in the development of atherothrombosis. Of particular importance is that CRP interacts with Fcγ receptors on cells of the vascular wall giving rise to the release of pro-thrombotic factors. The present review focuses on distinct sources of CRP-mediated ROS generation as well as the pivotal role of ROS in CRP-induced tissue factor expression. These studies provide considerable insight into the role of the oxidative mechanisms in CRP-mediated stimulation of pro-thrombotic factors and activation of platelets. Collectively, the available data provide strong support for ROS playing an important intermediary role in the relationship between CRP and atherothrombosis.

## Introduction

Mounting evidence indicates that atherosclerosis is a chronic inflammatory process, and further, that inflammation plays an important role in acute coronary syndromes (Davì and Patrono, 2007). Considerable data exists to support a causative role for reactive oxygen species (ROS) in these inflammatory states. Specifically, ROS generation triggers a cascade of events including inflammation, endothelial cell injury, blood coagulation, and thrombosis. Understanding the exact role played by ROS in vascular inflammation, however, is made difficult by observations that the generation of ROS appears to occur via a number of differing cell types and through varied mechanisms. Further, there appears to be a complex interplay between ROS and other inflammatory mediators, such as C-reactive protein (CRP) (Zeller and Sullivan, 1992; Irani, 2000; Venugopal et al., 2003; Qamirani et al., 2005; Ryu et al., 2007; Singh et al., 2007; Wu et al., 2008), whereby ROS not only induce production of the inflammatory mediator, but are produced in response to the mediator. Thus a vicious cycle can lead to the development of chronic inflammation.

Biologically active CRP, an acute phase reactant and member of the pentraxin family of plasma proteins, is composed of five identical subunits each with a molecular weight of approximately 23,000 kDa. The pentamer circulates as a discoid and annular-shaped complex. Recent studies, however, have shown that the pentamer can locally dissociate to give biological active monomers (mCRP) although evidence for circulating mCRP is not currently available (Bharadwaj et al., 1999; Khreiss et al., 2004). The site of synthesis of CRP is predominately hepatic although the localization of mRNA for CRP in tissues and atheromatous plaque has been used to suggest that local production could contribute to inflammation in vascular disease.

C-reactive protein is an accepted serum marker for inflammation (Järvisalo et al., 2002; Danesh et al., 2004; Emerging Risk Factors Collaboration et al., 2010). Further, a number of studies have confirmed that an elevated CRP is associated with an increased risk of ischemic vascular events, such as myocardial infarction (Zebrack et al., 2002; Khreiss et al., 2005; Singh et al., 2006). CRP has been detected in atherosclerotic lesions in human coronary arteries both at the protein and mRNA levels (Heinrich et al., 1995; Hak et al., 1999; Willeit et al., 2000; Blackburn et al., 2001). However, although the role of CRP has been argued to be involved in the development of atherosclerotic plaque size (Paul et al., 2004; Hirschfield et al., 2005; Trion et al., 2005), it is also well correlated with activation of the blood coagulation system and subsequently increasing the risk of thrombosis (Danenberg et al., 2003; Bisoendial et al., 2005). This may provide an explanation for why some studies using plaque size as an endpoint measure do not find a pathophysiological role of CRP.

As stated above, ROS generation plays a significant role in inflammatory processes, and the subsequent activation of pro-thrombotic factors and platelets by inflammatory mediators may also be a critical component of atherothrombosis. However, the molecular mechanisms responsible for inflammation-mediated disease processes are not clear, especially as it occurs in CRP-related atherothrombosis. Thus, the present manuscript will focus on studies examining CRP-mediated molecular mechanisms that link ROS generation to the activation of both thrombotic risk factors and platelets and subsequent vascular dysfunction. We also highlight the importance of ROS in linking CRP to the development of atherothrombosis. Specifically, we discuss evidence for (1) the role of ROS in atherosclerosis; (2) the role of CRP in vascular inflammation and atherosclerosis; (3) involvement of CRP in ROS generation and atherosclerosis; and (4) ROS and CRP in platelet activation and thrombus formation. Finally, we provide an integrated model linking CRP to the downstream generation of ROS via the FcyR and pro-atherothrombotic events in vascular cells including endothelial and smooth muscle cells, macrophages, and platelets. Therefore, we conclude that CRP is not simply a marker of inflammation, but also directly contributes to the pathogenesis of atherothrombosis.

## Role of ROS in Atherosclerosis

Considerable support exists for overproduction of ROS playing a pivotal role in the development of CVD and that this may occur through a number of mechanisms (Bouloumie et al., 1999; Sorescu et al., 2002; Guzik et al., 2006; Lee and Hirani, 2006). For example, superoxide anion, a major component of endogenous ROS, has been observed to accumulate in the walls of human coronary arteries with atherosclerosis. Further, it is well-known that elevated production of ROS is linked to the over expression of pro-inflammatory mediators, which themselves play a critical role in early steps of atherogenesis (Sorescu et al., 2002; Guzik et al., 2006; Lee and Hirani, 2006). ROS also contribute to the modification of protein structure and function. Thus, oxidized-LDL (ox-LDL) plays a key role in the development of atherogenesis in different cell types, including endothelial cells (Huang et al., 1999), vascular smooth muscle cells (VSMC) (Kataoka et al., 2001), and monocytes (Xu et al., 1999). Importantly, the presence of LDL oxidation can be observed *in situ* by the localization of specific ox-LDL antibodies within atherosclerotic lesions (Palinski et al., 1989; Ylä-Herttuala et al., 1989). In addition, oxidative stress alters the balance of vasodilator and vasoconstrictor mechanisms. Consistent with this, oxidative stress decreases NO bioavailability due to the breakdown of NO by ROS and the formation of highly reactive and damaging nitrosyl species that lead to endothelial dysfunction and eventually the development of atherosclerotic changes (Singh et al., 2007; Bonomini et al., 2008; Giacco and Brownlee, 2010; Hulsmans and Holvoet, 2010). Further, studies have shown that ROS can increase ET-1 production in cultured endothelial (Davì and Patrono, 2007) and VSMC (Irani, 2000), although, ROS does not appear to be the stimulus for ET-1 release during acute stress *in vivo* (Venugopal et al., 2003). Similarly, hydrogen peroxide (H_2_O_2_) has been shown to participate in the increased *in vivo* synthesis of constrictor prostaglandins (Singh et al., 2007). Collectively, the above information provides strong support for ROS contributing to the development of atherosclerosis and further information can be found in a number of recent reviews (Bonomini et al., 2008; Giacco and Brownlee, 2010; Hulsmans and Holvoet, 2010).

## Role of CRP in Vascular Inflammation and Atherosclerosis

C-reactive protein is traditionally considered as the prototypic marker of inflammation and is one of the strongest predictors of cardiovascular events that are currently available (Järvisalo et al., 2002). Accumulating evidence has demonstrated that CRP is both present in atherosclerotic plaques and that it may play an important role in promoting atherogenesis through the regulation of expression and release of inflammatory cytokines (Khreiss et al., 2005; Singh et al., 2006). Plasma CRP levels correlate poorly with atherosclerotic plaque burden in humans (Zebrack et al., 2002). In addition to its production by the liver (Gabay and Kushner, 1999), CRP has been reported to be produced by macrophages and smooth muscle cells (Dong and Wright, 1996; Kobayashi et al., 2003; Khera et al., 2006) and mRNA for CRP has been detected within human atherosclerotic plaque further supporting local synthesis (Jabs et al., 2003). However, doubt still exists as to whether CRP in the atherosclerotic lesions originates from the circulation or is locally synthesized by the vascular cells (Dong and Wright, 1996; Jabs et al., 2003; Kobayashi et al., 2003; Khera et al., 2006). Sun et al. (2005) using established animal atherosclerosis models, i.e., both cholesterol-fed and Watanabe heritable hyperlipidemic rabbits, have shown that CRP found in the atherosclerotic lesions was essentially derived from the circulation rather than synthesized *de novo* by vascular cells.

C-reactive protein has been shown to induce pro-inflammatory effects through overproduction of several pro-inflammatory mediators including monocyte chemoattractant protein-1 (MCP-1), intercellular cell adhesion molecule (ICAM), and vascular cell adhesion molecule-1 (VCAM-1). This CRP-dependent response has been observed in a number of human types such as endothelial cells, VSMC, and macrophages (Pasceri et al., 2000, 2001; Devaraj and Jialal, 2011). Additionally, CD40 ligand (CD40L) is expressed on the surface of platelets, T lymphocytes, and endothelial cells (Schönbeck and Libby, 2001; Lin et al., 2004) There is evidence that the CD40L level is a strong predictor of cardiovascular risk. The pro-inflammatory effects of CRP via CD40–CD40L signaling pathways also involved in the pathogenesis of atherosclerosis (Lin et al., 2004). Collectively these studies are consistent with the proposition that CRP may exert its pro-atherogenic effects through enhanced expression of pro-inflammatory mediators including both chemokines and adhesion molecules. Despite this, it continues to be debated whether CRP plays a causal role in the development of atherosclerosis, or is simply an important clinical marker of inflammation and cardiovascular risk (Zacho et al., 2008).

Recent experiments, crossing human CRP-transgenic mice into ApoE-knockout mice, demonstrated that CRP promotes, inhibits, or has no effect on atherosclerotic plaque growth (Paul et al., 2004; Hirschfield et al., 2005; Trion et al., 2005). The reason responsible for this discrepancy remains to be identified, but human CRP was present in the developing atheromas, including human and CRP-transgenic mice vascular lesions (Paul et al., 2004). Although there is apparent argument regarding the role of CRP in the risk of atherosclerosis, the positive results support the need to explore the exact mechanisms by which CRP might trigger the development of atherosclerotic lesions. Recent human studies demonstrated that elevated CRP levels more likely is a marker for the extent of atherosclerosis or for the inflammatory activity of atherosclerotic plaques, and effectively exclude that genetically elevated CRP cause CHD (Nordestgaard and Zacho, 2009).

The Justification for the Use of Statins in Primary Prevention: an Intervention Trial Evaluating Rosuvastatin (JUPITER) trial (Ridker et al., 2008, 2010), have reported the role of CRP as a biomarker of risk for cardiovascular disease and established it as a means of monitoring the impact of cholesterol-lowering therapy, not only in people with known risks, but also in asymptomatic individuals previously considered at average risk for myocardial infarction and stroke. The study emphasizes the importance of inflammation in cardiovascular disease and could result in changes to recommended cardio-protective practices and patient management. The effectiveness of statins in the JUPITER trial lower CRP levels as well as cholesterol, however, it is still argued whether statin drugs reduces the incidence of major cardiovascular events (Hingorani et al., 2009; Ridker et al., 2010).

A number of prospective epidemiologic studies have consistently demonstrated a strong association between CRP concentrations measured in initially healthy individuals and the risk of a first CHD event (Hingorani et al., 2009). There is considerable interest in whether CRP has a causal role in CHD whether CRP is merely a marker of underlying atherosclerosis (Pepys, 2008; Zacho et al., 2008). These arguments support the hypothesis that CRP increases the risk of CHD, not by promoting atherosclerotic plaque size, but rather by activating the blood coagulation system and increasing the risk of thrombosis.

## ROS and CRP in Atherosclerosis

As outlined above ROS overproduction is believed to be involved in the signal transduction processes leading to vascular inflammation and ultimately the progression of atherosclerosis (Basta et al., 2005; Thomas et al., 2008). Oxidative stress results in cell dysfunction and death. Major enzymes responsible for ROS generation in vascular cells include xanthine oxidase, cytochrome P-450, nitric oxide synthase (NOS), and NAD(P)H oxidase (Bonomini et al., 2008; Giacco and Brownlee, 2010; Hulsmans and Holvoet, 2010). CRP stimulates ROS production by various vascular cell types *in vitro*, including endothelial, smooth muscle, and monocyte/macrophages (Zeller and Sullivan, 1992; Irani, 2000; Venugopal et al., 2003; Qamirani et al., 2005; Ryu et al., 2007; Singh et al., 2007; Wu et al., 2008). CRP was co-localized with p22phox by VSMC, both *in vitro* and *in vivo*, an essential component of NADPH oxidase, which is an important source of ROS in vasculature (Kobayashi et al., 2003). CRP could activate NAD(P)H oxidase and upregulate ROS production in both endothelial cells and macrophages (Singh et al., 2005; Zhao et al., 2011). CRP promotes superoxide anion release from endothelial cells and reduces NO bioavailability by involvement of p22phox and p47phox (Singh et al., 2007). In porcine coronary arterioles, the treatment of CRP significantly increased NAD(P)H oxidase activity and this was mediated via the activation of mitogen activated protein kinase p38MAPK (Qamirani et al., 2005). Furthermore, accumulation of Ox-LDL in atherosclerotic lesions is a well-known event in the development of atherosclerosis. CRP promotes Ox-LDL uptake and matrix metalloproteinase induction through Fcγreceptor from macrophage and endothelial cells (Singh et al., 2008; Schwedler et al., 2009). Collectively, these findings support the suggestion that intracellular ROS generation is associated with CRP-associated atherosclerosis.

## ROS and CRP in Platelet Activation and Thrombus Formation

In response to blood vessel injury, platelets accumulate at the site of damage to form a clot. ROS play a key role in this process promoting platelet aggregation and thrombosis (Ikeda et al., 1994). Consistent with this role, antioxidants have been shown to reduce platelet aggregation (Przyklenk and Kloner, 1993). In addition, NO insufficiency, combined with ROS overproduction, predisposes to a platelet-dependent pro-thrombotic disorder by decreasing NO-dependent vasorelaxation and increasing platelet aggregation (Freedman et al., 1996; Keaney et al., 1996). FcγRIIa and FcγRIII are expressed on platelet, and ligand-binding of these receptors can directly activate platelets resulting in platelet aggregation. CRP has been reported to bind to the family of FcγRs in platelets including FcγRIIa and FcγRIII (Järvisalo et al., 2002). Previous studies have demonstrated that CRP inhibits platelet aggregation induced by a variety of agonists, including thrombin, platelet aggregating factor (PAF), and immunoglobulin (Fiedel and Gewurz, 1976; Filep et al., 1991; Cheryk et al., 1996; Filep, 2009). CRP also promotes platelet adhesion to endothelial cells and monocytes (Yaron et al., 2006; Danenberg et al., 2007), which is linked to causation of thrombosis. A previous study showed that stimulation of human neutrophils, monocytes, and platelets by modified CRP express a neoantigenic specificity (Potempa et al., 1988). Recently, Molins et al. (2008) showed that there was a facilitation of thrombus growth after platelet stimulation with monomeric CRP. The conformation of CRP (i.e., monomeric vs. pentameric) conceivably plays a role in the regulation of platelet aggregation (Bharadwaj et al., 1999; Khreiss et al., 2004). Activated platelets have been also shown to directly contribute to the conversion of pentameric CRP (pCRP) to the monomeric form (mCRP) (Devaraj et al., 2003, 2009; Eisenhardt et al., 2009). In fact, the conformation of CRP plays a different role in activation of molecular mechanisms. pCRP has been reported to inhibit insulin activation of endothelial NOS via FcγRIIB and Src homology 2 domain-containing inositol 5′-phosphate (SHIP-1), and activation of nuclear factor κB in monocytes, which may leads to endothelial dysfunction and release of pro-inflammatory cytokines (Filep, 2009; Tanigaki et al., 2009).

In addition to platelet-mediated actions, CRP has been linked to thrombosis, via direct effects on plasminogen activator inhibitor-1 (PAI-1), tissue plasminogen activator, and tissue factor activity in vascular cells (Danenberg et al., 2003; Singh et al., 2005; Ryu et al., 2007; Wu et al., 2008). CRP induces superoxide anion release tissue factor (Wang et al., 2005). PAI-1 is a marker of impaired fibrinolysis and atherothrombosis. CRP induces PAI-1 mRNA, antigen, and activity in endothelial cells, suggesting that CRP may be an atherothrombotic agent CRP induces PAI-1 mRNA, antigen, and activity in ECs (Devaraj et al., 2003). Pre-treatment with antioxidants such as pyrrolidine dithiocarbamate (PDTC), *N*-acetylcysteine (NAC) resulted in a marked attenuation of CRP-mediated tissue factor activity in human VSMC (Wu et al., 2008). Furthermore, CRP-induced FcγRIIa activation triggers NADPH oxidase 4 activation and subsequent ROS generation, which demonstrated the direct relationship between CRP and ROS via FcγR (Ryu et al., 2007; Zhao et al., 2011). *In vivo*, mice carrying a human CRP transgene have been shown to exhibit accelerated thrombosis and increased intimal hyperplasia following vascular injury (Danenberg et al., 2003; Wang et al., 2005). Furthermore, injection of highly purified CRP into humans activates the inflammation and blood coagulation system (Bisoendial et al., 2007). Therefore, CRP may trigger clinical ischemic events by promoting thrombosis. The consequences of CRP and ROS in platelet activation and thrombus formation, as well as atherosclerosis, are key features of atherothrombosis.

## Conclusion

**Figure 1:**
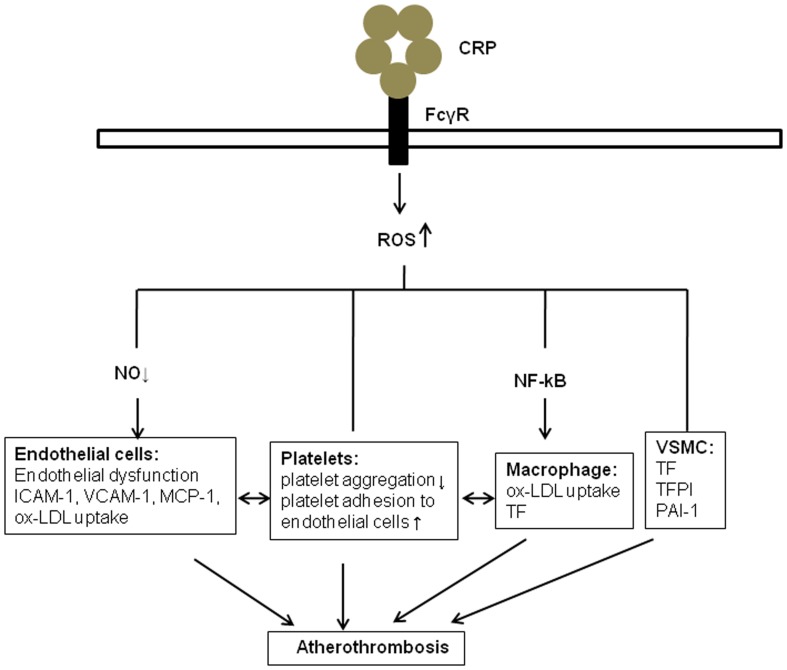
**The role of ROS in CRP and atherothrombosis**. CRP promotes the generation of reactive oxidant species (ROS) through binding to ECs, platelets, monocytes, and VSMC via specific Fcγ receptors. Several potential mechanisms are involved, including the inhibition of endothelial nitric oxide synthase, activation nuclear factor κB in monocytes, and release of extrinsic and intrinsic coagulation factors in vascular smooth muscle cells (VSMC). These molecules and cell–cell interactions, in turns, may contribute to endothelial dysfunction, release of cytokines. Ox-LDL, Oxidized low-density lipoprotein; VSMC, Vascular smooth muscle cell; MCP-1, monocyte chemoattractant protein-1; ICAM-1, intercellular adhesion molecule-1; VCAM-1, vascular adhesion molecule-1; TF, tissue factor; TFPI, tissue factor pathway inhibitor; PAI-1, plasminogen activator inhibitor-1.

In summary, the presence of CRP in atherosclerotic lesions, likely directly contributes to ROS generation in association with family of FcγRs in platelets, and is linked to the regulation of pro-thrombotic events, including activation of blood platelets, the extrinsic blood coagulation cascade, and the fibrinolytic system (Figure 1). ROS generation induced by CRP in vascular cells is associated with the production of tissue factor. Further studies are necessary to define more precisely the pro-thrombotic functions of CRP-related ROS generation. In addition, more research is needed in order to determine if specifically inhibiting CPR-modulated ROS would inhibit arterial thrombosis.

## Conflict of Interest Statement

The authors declare that the research was conducted in the absence of any commercial or financial relationships that could be construed as a potential conflict of interest.
